# Point-of-care ultrasound in patients with suspected deep vein thrombosis (DVT)

**DOI:** 10.1186/1757-7241-21-S2-A39

**Published:** 2013-09-09

**Authors:** Mohammad Al hashimy, Kim Hvid Benn Madsen, Thomas Andersen Schmidt

**Affiliations:** 1 Emergency Department, Holbaek Hospital , Denmark; 2 Emergency Department, Holbaek Hospital , Denmark; 3 Emergency Department, Holbaek Hospital , Denmark

## Background

Suspicion of Deep vein thrombosis (DVT) is a frequent cause of presentation in emergency departments (EDs). Traditionally at Holbaek University Hospital, patients presenting with suspected lower-extremity DVT are commonly assessed and treated as out-patients in the Quick Diagnostic Unit (QDU), a part of our Emergency Department. The patients undergo D-dimer testing and Wells score followed by ultrasound (US) only if the D-dimer is positive, or the patient is judged clinically to have DVT (Wells score>2). Unfortunately, the limited availability of radiologist-performed ultrasound outside banker’s hours delay the diagnosis by more than 24 hours and may expose the patient to inappropriate anticoagulation treatment. The safety, ease of use, rapid time of diagnosis, low cost and accessibility makes bedside ultrasound for DVT especially useful for emergency physicians.

The aim of this pilot study is to assess the time-to-diagnosis and the accuracy of emergency physician performed bedside ultrasound (EPUS) in the detecting of pathological findings (Hematoma, Baker's cyst and Thrombosis), in comparison with the traditional settings involving a radiologist-performed ultrasound.

## Methods

10 patients with clinically suspected proximal DVT attending our QDU were included in our pilot study. All patients enrolled underwent whole-leg US performed by an emergency-physician and a radiologist.

## Results

10 patients were enrolled in this pilot study. The EPUS findings were normal in 7 patients (70%), abnormal in 3 patients (30%).

All normal test results were confirmed by the radiologist, and 3 patients with abnormal findings on EPUS examination were subsequently diagnosed as having distal DVT or superficial thrombophlebitis.

The mean time-to-diagnosis of EPUS was 2:45 h (range 00:45 to 04:12h) compared to the mean time-to-diagnosis performed by a radiologist of 27:23 h (range 04:30 h to 71:03 h), p < 0.002.

The US performed by an emergency physician had a sensitivity of 100 % and specificity of 100 %.

## Conclusion

Our findings suggest that EPPU may be useful in excluding pathological findings in patients with suspected DVT, and may allow rapid discharge and avoiding unnecessary anticoagulant treatment. Future prospective studies are warranted to confirm these findings. This study will continue over the next few months.

